# Evaluation of the Variability of the ORF34, ORF68, and MLST Genes in EHV-1 from South Korea

**DOI:** 10.3390/pathogens10040425

**Published:** 2021-04-02

**Authors:** Hyung-Woo Kang, Eun-Yong Lee, Kyoung-Ki Lee, Mi-Kyeong Ko, Ji-Young Park, Yeon-Hee Kim, Kyunghyun Lee, Eun-Jin Choi, Jongho Kim, ByungJae So, Choi-Kyu Park, Hye-Young Jeoung

**Affiliations:** 1 Animal and Plant Quarantine Agency, 177 Hyeoksin 8-ro, Gimcheon-si 39660, Korea; guddn1812@korea.kr (H.-W.K.); agdragon@korea.kr (E.-Y.L.); naturelkk@korea.kr (K.-K.L.); mkk80@korea.kr (M.-K.K.); jijipy@korea.kr (J.-Y.P.); vetyh@korea.kr (Y.-H.K.); mylovehyun@korea.kr (K.L.); choiej@korea.kr (E.-J.C.); whdgh2339@korea.kr (J.K.); bjso@korea.kr (B.S.); 2 College of Veterinary Medicine & Animal Disease Intervention Center, Kyungpook National University, Daegu 41566, Korea; parkck@knu.ac.kr

**Keywords:** EHV-1, ORF30, ORF33, ORF34, ORF68, MLST, phylogeny

## Abstract

Equine herpesvirus-1 (EHV-1) is an important pathogen in horses. It affects horses worldwide and causes substantial economic losses. In this study, for the first time, we characterized EHV-1 isolates from South Korea at the molecular level. We then aimed to determine the genetic divergences of these isolates by comparing them to sequences in databases. In total, 338 horse samples were collected, and 12 EHV-1 were isolated. We performed ORF30, ORF33, ORF68, and ORF34 genetic analysis and carried out multi-locus sequence typing (MLST) of 12 isolated EHV-1. All isolated viruses were confirmed as non-neuropathogenic type, showing N752 of ORF30 and highly conserved ORF33 (99.7–100%). Isolates were unclassified using ORF68 analysis because of a 118 bp deletion in nucleotide sequence 701–818. Seven EHV-1 isolates (16Q4, 19R166-1, 19R166-6, 19/10/15-2, 19/10/15-4, 19/10/18-2, 19/10/22-1) belonged to group 1, clade 10, based on ORF34 and MLST analysis. The remaining 5 EHV-1 isolates (15Q25-1, 15D59, 16Q5, 16Q40, 18D99) belonged to group 7, clade 6, based on ORF34 and MLST analysis.

## 1. Introduction

Equine herpesvirus-1 (EHV-1), a major pathogen infecting horses, can have devastating effects, causing severe economic burden in the horse industry worldwide [1]. EHV-1 belongs to the subfamily *Alphaherpesvirinae* and family *Herpesviridae*. It has a linear, double-stranded DNA genome of approximately 150 kbp, containing 80 open reading frames (ORFs), four of which are duplicated, and consists of long and short unique regions (UL and US, respectively), and the former is flanked by a small inverted repeat (TRL/IRL) and the latter by a large inverted repeat (TRs/IRs) [2]. Several EHV-1 infections are sub-clinical, but the virus can also cause respiratory diseases of varying severity, abortion, neonatal death, or neurological disease, referred to as equine herpesvirus myeloencephalopathy (EHM) [3]. Although the neurological form of the disease is less common than abortion or respiratory disease, EHM appears to have increased in some parts of the world over the past 10–15 years, causing concerns among horse owners and veterinarians because it can result in fatalities [4]. EHV-1 is spread by saliva and nasal discharge as well as aborted fetuses, placentas, or placental fluids [5,6,7,8].

Previous studies have primarily analyzed ORF30 and ORF33 (gB) genes related to pathogenicity [9,10,11,12]. The ORF30 gene, which encodes the DNA polymerase gene, is considered a marker of pathogenicity because its potential to cause neuropathogenicity is significantly higher in EHV-1 strains that carry a single nucleotide polymorphism (SNP) at the nucleotide position 2254 and it causes a substitution of asparagine (N) to aspartic acid (D) at amino acid position 752 in the catalytic subunit of the viral DNA polymerase [10,13]. Therefore, EHV-1 N752 is referred to as a non-neuropathogenic genotype, and D752 is a neuropathogenic genotype [14]. The ORF33 gene, encoding glycoprotein B (gB), possesses a conserved region frequently used as a target for diagnostic PCR protocols [15,16]. Recently, genetic studies include the determination of whole-genome sequences for two well-characterized strains, Ab4 and V592 [2,10]. Strain Ab4, isolated from a quadriplegic gelding, was associated with severe neurological disease and frequent abortion [1]. In contrast, strain V592, isolated from a fetus during a large abortion storm, appears to be less virulent on experimental infection, resulting in low levels of viremia, few cases of abortion, and no neurological disease [10].

Comparison of the sequence of the neuropathogenic strain Ab4 with that of the abortigenic strain V592 identified 43 amino acid residue differences distributed among 31 ORFs [10,17]. Of these, ORF68, which encodes a non-essential, membrane-associated virion component, was shown to display genetic heterogeneity and was developed as a target for classifying the field isolates into six groups [10]. This method was subsequently used to compare isolates from different geographical regions [10]. However, there have been conflicting conclusions regarding the usefulness of this approach for molecular tracking of EHV-1 in other countries [15]. ORF34, which encodes the V32 protein, was the most variable in this viral collection and was also classified into distinct groups by SNPs in their genomes [10] and allowed the identification of at least 12 groups [16]. Recently, a multi-locus sequence typing (MLST) analysis approach, based on sequencing heterologous regions in 26 ORFs, which has diverged into 13 distinct UL clades, proved to be a more comprehensive method of strain typing than only ORF68 sequencing [15].

EHV-1 from aborted fetuses was first reported in South Korea in 1979 [18]. Subsequently, several surveys of EHV-1 have been reported in several clinical samples, primarily from aborted fetuses [19], and only one case report of EHV-1 related myeloencephalopathy with the D752 genotype was described [20]. However, the sequence of ORF30 is absent in GenBank. Unfortunately, there is limited information regarding the molecular variability in EHV-1 in South Korea. The present study aimed first to analyze and classify 12 EHV-1 isolates collected in South Korea over the past 5 years at the molecular level and subsequently use these isolates to investigate the genetic divergences of EHV-1 in comparison with sequences available in genetic databases and bibliographies.

## 2. Results

### 2.1. EHV-1 Identification and Virus Isolation

EHV-1 was detected in 12 equine samples (3.55%, 12/338). The 134 horses with neurological symptoms did not have EHV-1 detected, and 51 of these samples had several parasites detected, such as strongyle and *Halicephalobus gingivalis* [21]. There were two aborted fetuses in 2015, three in 2016, one in 2018, and four lung samples and two nasal swabs from cases with respiratory symptoms in 2019. Two strains were isolated from the same farm: 19R66-1 and 19R66-6. EHV-1 positive samples were isolated from RK-13 cells. The 12 samples were confirmed by ORF33 specific real-time PCR [9] (Table 1). The virus line had a visible CPE (rounding, clustering and lysis) 3 days post-inoculation. EHV-1 viruses were isolated from positive samples using RK-13 cells.

### 2.2. ORF30 Sequence Analysis

For ORF30 analysis, the 12 EHV-1 isolates were detected by nested PCR with 485 bp and 329 bp (data not shown). The sequences were deposited in GenBank (accession numbers MT675191–MT675202, Table S1). None of the analyzed sequences belonged to the D752 neuropathogenic genotype. Another SNP was obtained in this study and was found at G2968A (E990K) in seven samples (16Q4, 19R166-1, 19R166-6, 19/10/15-2, 19/10/15-4, 19/10/18-2, and 19/10/22-1), in comparison with the reference strain Ab4.

### 2.3. ORF33 Sequence Analysis

ORF33 was verified by nested PCR with 1440 bp and 770 bp (data not shown). They were deposited in GenBank (accession numbers MT559576-MT559587, Table S1). ORF33 was highly conserved (99.7–100% homology) in the 12 EHV-1 isolates, with few SNPs. In comparison with the reference strain Ab4, a synonymous mutation was observed in two samples (19/10/15-2, 16Q5). There were changes to A1508G (K503R) at 19/10/15-2 and C1567T (L523F) at 16Q5.

### 2.4. ORF68 Sequence Analysis

Comparison of the V592 sequence with that of Ab4 resulted in the identification of a polymorphic region of ORF68 that was found to be particularly useful for grouping isolates as this locus displays several SNPs within a relatively short region. The nucleotide sequence of the Ab4 strain as a member of EHV-1 group 1 served as a basis for comparing nucleotide changes [10]. PCR products from the 12 EHV-1 isolates were obtained from the ORF68 region of the genome, including the approximately 600 bp-long polymorphic segments, which were sequenced and aligned to distinguish SNPs. The sequences were deposited in GenBank (accession numbers MT940243–MT940254, Table S1), and these SNPs are presented in Table 2.

### 2.5. ORF34 Sequence Analysis

**Figure 1:**
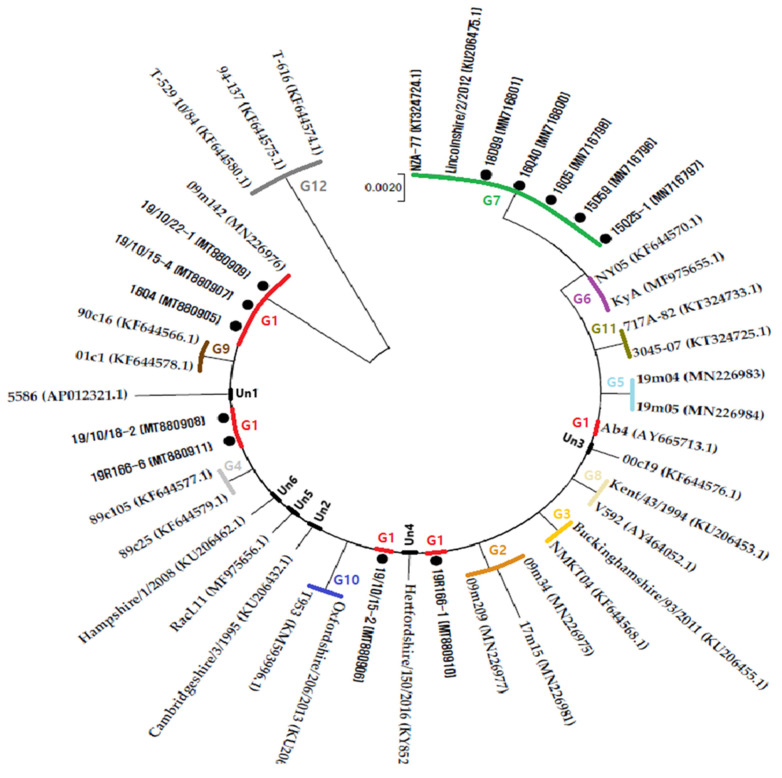
The evolutionary history of ORF34 sequences was obtained using the neighbor-joining method with bootstrap test with 1000 replicates. The evolutionary distances were computed using the Tamura 3-parameter method and are in the units of the number of base substitutions per site. Sequences obtained in this study are marked with a circle (●). The letter “G” followed by a number indicates the number of the group where sequences are located. The letters “Un” followed by a number indicates the sequences not located in any group.

The sequence analysis of ORF34 could be categorized into twelve groups [13]. The 12 EHV-1 isolates belonged to group 1 (16Q4, 19R166-1, 19R166-6, 19/10/15-2, 19/10/15-4, 19/10/18-2, and 19/10/22-1) and group 7 (15Q25-1, 15D59, 16Q5, 16Q40, and 18D99) (Figure 1). The sequences were deposited in GenBank with accession numbers MT880905-MT880911 for group 1 and MN716796-MN716798, MN716800-MN716801 for group 7 (Table S1). Analysis of the ORF34 sequences of group 1 compared to those of group 7 showed an SNP at T156G (Q52H) and one non-synonymous mutation, C303A (Table 3), which were newly confirmed in this study. A simplified tree including only selected sequences representative of each observed nucleotide variation is shown in Figure 1.

### 2.6. Phylogeny and Multi-Locus Sequence Analysis

**Figure 2:**
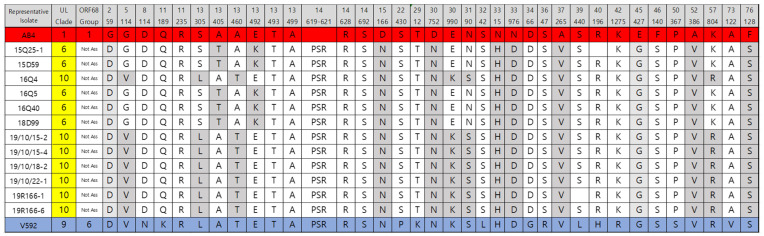
Multi-locus sequence analysis of representative equine herpesvirus-1 (EHV-1) isolates in this study using 38 amino acid differences in 26 open reading frames (ORFs). The ORF variable area is highlighted in gray.

**Figure 3:**
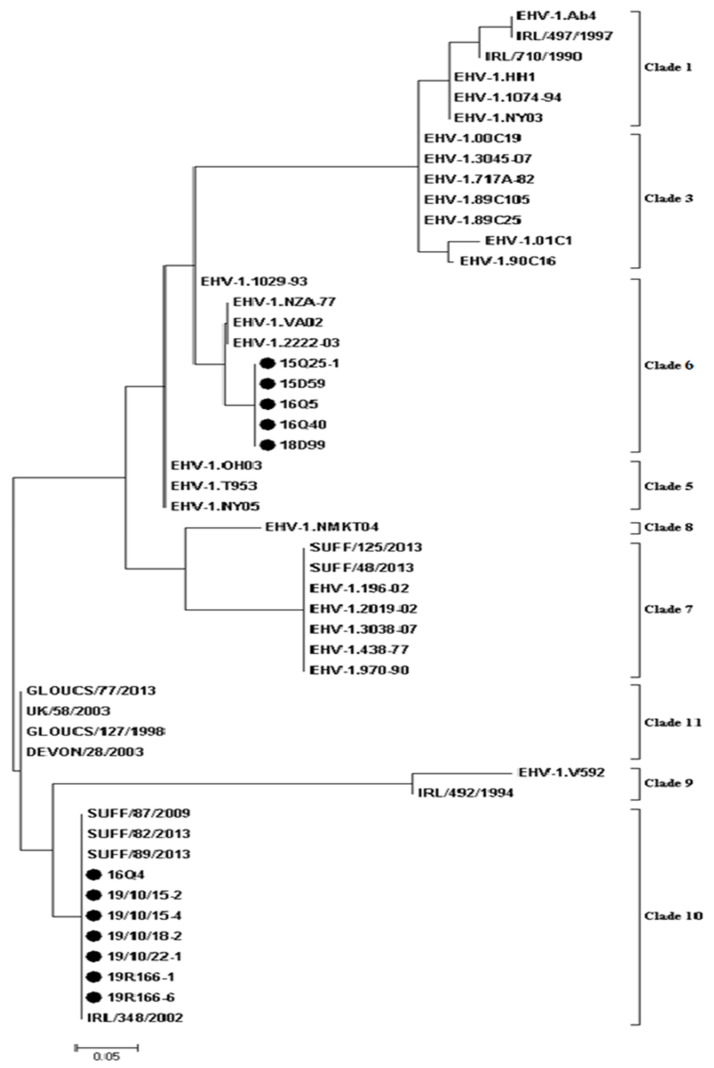
Maximum likelihood phylogenetic tree of amino acid sequences based on the Jones Taylor Thornton (JTT) matrix-based model. The tree is based on the alignment of the artificial peptide derived by multi-locus sequence typing of 12 equine herpesvirus-1 (EHV-1) isolates sequenced in this study and EHV-1 strains obtained from GenBank with known U_L_ clade grouping. EHV-1 U_L_ clades are indicated by bars and are numbered according to the key. Bootstrap values after 1000 replications are indicated at major nodes. Dots indicate Korean isolated strains.

Phylogenetic analysis was performed using an artificial peptide consisting of concatenated amino acids of UL and US based on 31 non-synonymous substitutions between Ab4 and V592 [1]. MLST analysis for the 12 EHV-1 strains is shown in Figure 2 and Figure 3. Five strains (15Q25-1, 15D59, 16Q5, 16Q40, and 18D99) were grouped as UL clade 6, and seven strains (16Q4, 19R166-1, 19R166-6, 19/10/15-2, 19/10/15-4, 19/10/18-2, and 19/10/22-1) as UL clade 10.

## 3. Discussion

EHV-1 is a World Organization for Animal Health (OIE) listed disease that must be notified to OIE to ensure safe international trade in horses [22]. Considering the potential economic and emotional impact of EHV-1 infections, it would be beneficial to understand the molecular evolution of these viruses, facilitated by tracking the sources of EHV-1 during an outbreak and developing effective control and prevention strategies. Unfortunately, limited genetic data are available in Korea. To date, only ORF30 and ORF33 have been analyzed [19,20,23]; however, these sequences are not present in databases.

In this study, various clinical equine samples were collected in Korea and analyzed for molecular characterization of 12 EHV-1 isolates. ORF30, ORF33, ORF34, and ORF68 of all isolates were sequenced, and the genetic information was deposited in GenBank. Unfortunately, we could not detect EHV-1 in 134 samples from horses with neurological symptoms; however, several reports demonstrated that nucleotide substitution N752 of ORF30 is not the only determinant of neurological disease [24]. EHV-1 N752 genotype viruses are more commonly associated with abortion, responsible for 15~26% of the EHM outbreak [25]. All isolated viruses belonged to the ORF30 N752 genotype, indicating non-neuropathogenic EHV-1 in Korea [19,23,26]. Although the ORF30 D752 genotypes (3/55, 5.5%) were reported in 2014 in Korea [18], this neurological genotype may not be common in Korea. Recently, similar studies were reported in other countries, and the prevalence of the neuropathogenic genotype was extremely low in Japan (2.7%), Australia (3%) and Argentina (7.4%) [23]. In particular, neuropathogenic cases were extremely rare in Korea [16]. Furthermore, this study found an additional SNP at G2968A (E990K) in seven samples by comparing with the reference strain Ab4. Although this SNP was previously reported as not associated with pathogenicity, data are insufficient to predict the impact of the substitutions on protein activity [22].

The homology of ORF33 in this study was 99.7–100% with a few SNPs in two isolated strains (19/10/15-2, 16Q5), while the other 10 EHV-1 isolates showed sequence identity with the ORF33 sequence of Ab4. Our data suggest that this sequence of the ORF33 gene is generally highly conserved and is a good target for diagnostic methods, although some SNPs were observed. A few solitary changes in sequence usually do not affect diagnostic sensitivity, and continuously monitoring these changes is important to avoid false-negative results of EHV-1 diagnosis.

Since 2006, the ORF68 polymorphic region has been used as a putative molecular marker in epidemiological studies. It has commonly been used for 6 groups of EHV-1 isolates in different countries, including Australia, Japan, and Poland [3,27,28]. Group 4 and group 5 genotypes were reported to be predominant in Europe, Africa, and North America, and the group 2 genotype was reported in Japan [15]. Following the original proposal of the 6 groups of ORF68, more SNPs have been described, and new groups have been proposed [1,3,28]. ORF68 sequence analysis of the 12 EHV-1 isolates revealed that they were unclassified according to the groups originally described by Nugent et al. [10] because these isolates showed a 118 bp deletion of the nucleotide sequence 701–818, resulting in a shorter amino acid sequence. Indeed, it was the same deletion found in KyA (MF975655) and Racl11 (MF975656), isolated in the USA, which were unclassified [24]. In the present study, all isolates had an infrequent deletion of 118 (701–818) bp in the ORF68 gene, which was unclassified. The same results were obtained for the unassigned group in KyA, RacL11, and Italian isolates [16,29]. Although ORF68 sequences of the 12 EHV-1 isolates were very similar to KyA and Racl11, these have SNPs at A236C, G689T, and C690T compared to KyA and RacL11 (Table 2). The 09m142 (MN226987), which was isolated from Italy, also showed the A629 (H210) variation that was present in the 12 EHV-1 isolates. In particular, 7 strains (16Q5, 19R166-1, 19R166-6, 19/10/15-2, 19/10/15-4, 19/10/18-2, and 19/10/22-1) demonstrated 100% homology with 09m142 (MN226987) in nucleotide sequence 236–825 of ORF68. Similar to the results of the studies performed in Hungary and Poland, this suggests that ORF68 is not a suitable global marker [1,3]. However, this type of strain variation has been demonstrated to be a useful adjunct to epidemiological data when investigating disease outbreaks on multiple premises [10,26,30,31]. Furthermore, the presence of the 118 (701–818) bp deletion in EHV-1 strains from other geographical areas and the pathogenic properties of isolates with this deletion should be thoroughly evaluated [16,27].

Studies have confirmed that ORF34 exhibits the highest sequence variability, which should be used to determine whether ORF34 can be a useful marker [17]. Via ORF34 analysis, the 12 groups were identified and named 1 to 12, in which group 1 includes the reference strain Ab4 and group 12 includes isolated strains from zebra, onager, and Thomson’s gazelle [28]. Here, a complete analysis of the ORF34 sequences available in GenBank or reported in bibliographies showed that some SNPs were repeated in strain groups [15,17,32]. The 12 EHV-1 isolates in Korea were located in groups 1 and 7. Group 1 (16Q4, 19R166-1, 19R166-6, 19/10/15-2, 19/10/15-4, 19/10/18-2, and 19/10/22-1) showed an ORF34 sequence identical to the sequence of the reference strain Ab4. Group 7 (15Q25-1, 15D59, 16Q5, 16Q40, and 18D99) revealed new SNPs that have not been reported (Table 3). ORF34 studies suggest that the ORF34 protein is required for optimal EHV-1 replication in cultured cells during early infection [33]. The impact of different ORF34 gene mutations on viral replication is unknown. As limited investigations have been carried out thus far, we can speculate that more SNPs will be found in the ORF34 gene, new groups will be described, and the function of the molecular maker for ORF34 will be discovered [16].

MLST analysis provides a more global view of EHV-1 strain evolution as it takes into account 37 loci in 26 different ORFs, and this approach was first used by Nugent et al. [10] and extended in other studies to allow comparison of recently proposed U_L_ clades [17]. In this study, MLST analysis of the 12 isolated EHV-1 strains was performed using non-synonymous substitutions between the EHV-1 reference strains, Ab4 and V592. Our results showed that clade 6 and clade 10 of 12 isolated EHV-1 were observed. There were correlations between the results of ORF34 and MLST analyses. The five EHV-1 isolates (15Q25-1, 15D59, 16Q5, 16Q40, and 18D99) were found to belong to group 7 and clade 6 using ORF34, and MLST analysis, respectively, and the 7 EHV-1 isolates (16Q4, 19R166-1, 19R166-6, 19/10/15-2, 19/10/15-4, 19/10/18-2, 19/10/22-1) were found to belong to group 1 and clade 10 using ORF34 and MLST analysis, respectively. Clade 6 of EHV-1 from the UK, United States, New Zealand, Australia, and clade 10 of EHV-1 from France and Belgium were reported [17]. EHV-1 from Japan belonged to clades 1 and 3 [34,35].

Genotyping studies on EHV-1 are extremely limited in Korea; however, our results suggested a possible co-circulation of two types of EHV-1 (groups 1 and 7 for ORF34 analysis, clades 6 and 10 for MLST analysis). EHV-1 in Korea may independently circulate to Asian countries such as Japan, closely related to Europe, the USA, and Oceania. There was, however, no information on the circulation of EHV-1 strains before 2015 and little information on the genetic diversity of EHV-1 in Korea and Asia. Therefore, there is a need for more information on several EHV-1 isolates in Korea through retrospective studies, and more data will have to be obtained via a collection of clinical samples. Additionally, there is a need for more genetic information on EHV-1 strains in Asian countries such as China.

Further studies of genetic diversity of EHV-1 will corroborate some premises as the source of virus will assist in implementation of targeted movement restriction, quarantine and other control measures. The contribution of genetic characterization to our understanding of viral pathogenesis, the development of diagnostics, and the predictions of the likely outcome of disease spread will increase in the future.

## 4. Materials and Methods

### 4.1. Sample Collection

A total of 273 samples were obtained from whole equine bodies from the Animal and Plant Quarantine Agency (APQA) to diagnose horse diseases from 2015 to 2019 throughout all seasons. The samples were collected across all Korean provinces geographically and included mainly clinical signs of EHV-1 such as abortion and respiratory symptoms [36]. Following autopsies of most horse samples, the tissue types submitted for investigation varied but typically included brain, spleen, liver, lung, heart, kidney, placenta, blood samples, nasal swabs, and genital swabs. From the 273 samples collected, it was found that 134 horses had neurological symptoms, 22 had respiratory symptoms, 15 were aborted fetuses, and 102 had other clinical symptoms such as loss of vigor, decreased appetite, gonarthritis, and emaciation. Additionally, for the detection of EHV-1, 65 samples were randomly collected in 2019 (9 nasal swabs with respiratory symptoms and 7 genital swabs with infertility from farms, 2 whole blood samples from farms, and 47 lung tissue samples from an abattoir in Jeju). Data on sex, age, breed, and clinical signs were recorded for blood samples, nasal swabs, and genital swabs, whereas the lung tissue samples represented individual horses with no additional data recorded. Eighty-two horse samples were submitted in 2015, 75 in 2016, 13 in 2017, 5 (2 tissue samples and 3 blood samples) in 2018, and 163 in 2019.

### 4.2. DNA Extraction and EHV-1 Identification

For DNA extraction from swabs and tissue samples, approximately 25 mg of tissue was ground in 2 mL of a serum-free minimum essential medium α (Gibco, UK) solution by using a homogenizer, and prepared by centrifuging the whole sample at 3500 rpm for 10 min [10].

DNA was extracted from homogenized clinical samples using the Intron^®^ Patho Gene-spin™ DNA/RNA Extraction Kit (iNtRON Biotechnology, Seongnam, Korea) according to the manufacturer’s instructions. For detection of EHV-1, ORF33 specific real-time PCR was performed [9]. Two microliters of the DNA extract were applied in the PCRs.

### 4.3. Virus Isolation

To isolate EHV-1, rabbit kidney (RK-13) cells were cultured in Dulbecco’s modified Eagle’s medium, DMEM (Gibco, Grand Island, NY, USA) supplemented with 10% fetal bovine serum, FBS (Gibco, Grand Island, NY, USA) and 1% antibiotic-antimycotic solution (Gibco, Grand Island, NY, USA).

Cells were observed daily and microscopically for the appearance of the virus. They were incubated at 37 °C in 5% (*v*/*v*) CO_2_ until 70% of cells showed cytopathic effect (CPE) [17,27].

All isolated EHV-1 samples were stored at −70 °C.

### 4.4. ORF30, ORF33, and ORF68 Sequence Analysis

Gene sequencing and analyses of ORF30, ORF33, and ORF68 of 12 isolated EHV-1 strains were performed. Nested PCR was carried out to detect ORF30; 30-1s (5′-TACCCAAGCATTATCCAG-3′) and 30-1sr1 (5′-GATAACCCTGACGGAGTAAG-3′) were the first set of PCR primers, and 30-2s (5′-GAGAAGACCTTTCAGCGAC-3′) and 30-2sr1 (5′-CTCAGCAGTCATAACGAAC-3′) were the second set [10]. Amplification conditions of both first and second round PCRs were 94 °C for 4 min, 40 cycles at 94 °C for 30 s, 60 °C for 30 s and 72 °C for 1 min, with a final extension at 72 °C for 10 min followed by refrigeration at 4 °C. The PCR products, 485 bp and 329 bp, were amplified and sequenced.

For amplification of ORF33 by nested PCR, EHV-1(gB) F (5′-TCTACCCCTACGACTCCTTC-3′) and EHV-1(gB) R (5′-ACGCTGTCGATGTCGTAAAACCTGAGAG-3′) were the first set of PCR primers, and EHV-1(gB) nF (5′-CTTTAGCGGTGATGTGGAAT-3′) and EHV-1(gB) nR (5′-AAGTAGCGCT TCTGATTGAGG-3′) were the second set [22].

Amplification conditions of both first and second round PCRs were 94 °C for 4 min, 40 cycles at 94 °C for 30 s, 60 °C for 30 s and 72 °C for 1 min, with a final extension at 72 °C for 10 min followed by refrigeration at 4 °C. The PCR products, 1440 bp and 770 bp, were amplified and sequenced.

Two new primers (ORF68-F1/ORF68-R1 and ORF68-F2/ORF68-R2) were used in this study to amplify ORF68, ORF68-F1 (5′-AGTTGTTACAGTTGTCGACA-3′) and ORF68-R1 (5′-GTCGACCACCGCGTAAATCA-3′), ORF68-F2 (5′-AGATGAGTTTCATAAATCTT-3′) and ORF68-R2 (5′-TTTTGCAAGGCAAGAAACCA-3′).

Amplification conditions of both first and second round PCRs were 94 °C for 5 min, 45 cycles at 94 °C for 1 min, 55 °C for 1 min and 72 °C for 1 min, with a final extension at 72 °C for 7 min followed by refrigeration at 4 °C.

All samples were analyzed using the multiple sequence alignment program BioEdit (v. 7.0.5.3). The sequences were aligned with those available in public databases, and SNPs were investigated.

### 4.5. ORF34 Sequence Analysis and Phylogeny

Nested PCR was performed using the method by Silvia Preziuso et al. [16]. The first primer set was 1058F (5′-GGCCCCAAGGATATTTAAGC-3′), and 1893R (5′-GTTTGAGGCGGTTACGTCAG-3′) and 1090Fi (5′-CCGAGGTTTCATCCTCATTC-3′) and 1784Ri (5′-GCGGACATATTCGTGTCTCA-3′) were used for the second PCR. Amplification conditions of both first and second round PCRs were 94 °C for 5 min, 45 cycles at 94 °C for 1 min, 58 °C for 1 min and 72 °C for 1 min, with a final extension at 72 °C for 7 min followed by refrigeration at 4 °C. The PCR products, 855 bp and 714 bp, were amplified and sequenced. Results of sequence alignments were corrected using BioEdit, and phylogeny analysis was performed with MEGA (v. 7.0.26) using the neighbor-joining method based on the Tamura 3-parameter distance model. Aligned sequences were analyzed using a similarity matrix. The stability of the acquired phylogenetic tree was assessed by 1000 replicate bootstrap analysis.

### 4.6. MLST Analysis

For multi-locus sequencing analysis, 37 loci of 26 ORFs were analyzed based on non-synonymous changes identified between different published data containing Ab4 and V592 protein-coding positions, as reported by Garvey et al. [10,15,22]. Comparative analysis of predicted partial amino acid sequences was performed using nucleotide sequences of individual ORFs for each isolate and translated using ClustalW implemented in BioEdit [15]. To identify the variable position and to group isolates in accordance with Nugent et al. [10,32,37,38,39], the sequence was arranged using Ab4 (GenBank Accession number AY665713.1) and V592 (GenBank Accession number AY464052.1) strains as references. Both phylogenetic trees were analyzed using the Maximum Likelihood and Jone Taylor Thornton models.

## Figures and Tables

**Table 1:** Summary of equine herpesvirus-1 (EHV-1) isolates in this study.

Year	Strain ID	Virus Source	Disease Type	Breed	Symptoms	Region
	(Name/Location)					
2015	15Q25-1	Aborted fetus	Abortion	Thoroughbred	Aborted fetus	Sangju-si
	15D59	Aborted fetus	Abortion	Thoroughbred	Aborted fetus	Icheon-si
2016	16Q4	Aborted fetus	Abortion	Hanoverian	Aborted fetus	Gunsan-si
	16Q5	Aborted fetus	Abortion	pony	Aborted fetus	Gunsan-si
	16Q40	Aborted fetus	Abortion	pony	Aborted fetus	Pyeongtaek-si
2018	18D99	Aborted fetus	Abortion	Unknown	Aborted fetus	Pyeongtaek-si
2019	19R166-1	Nasal swab	Respiratory disorders	Thoroughbred	Respiratory disease	Jangsu-gun
	19R166-6	Nasal swab	Respiratory disorders	Thoroughbred	Respiratory disease	Jangsu-gun
	19/10/15-2	Lung	Slaughter	Unknown	Abattoir (unknown)	Jeju Island
	19/10/15-4	Lung	Slaughter	Unknown	Abattoir (unknown)	Jeju Island
	19/10/18-2	Lung	Slaughter	Unknown	Abattoir (unknown)	Jeju Island
	19/10/22-1	Lung	Slaughter	Unknown	Abattoir (unknown)	Jeju Island

**Table 2:** Nucleotide sequence alignment of ORF68 of the 12 unassigned Korean EHV-1 strains, using strain Ab4 as a reference.

Accession No.	Group	236	336	344	620	626	629	633	689–690	701	710	713	719	738	739	743	755	783	818	825
Ab4 (AY665713.1)	1	C	C	G	C	T	G	A	TT	G	T	C	G	G	G	C	C	G	G	C
US85_1_1 (DQ172400.1)	1	*	.	.	.	.	.	.	..	.	.	.	.	.	GG	.	.	.	.	.
AR85_1_1 (DQ172310.1)	1	*	.	.	.	.	.	.	..	.	.	.	.	-	-	.	.	.	.	.
US89_1_1 (DQ172408.1)	2	*	.	.	.	.	.	.	..	.	.	.	.	.	.	T	.	.	.	.
US79_1_1 (DQ172394.1)	2	*	.	.	T	.	.	.	..	.	.	.	.	.	.	.	.	.	.	G
AR79_1_1 (DQ172309.1)	2	*	.	.	.	.	.	.	..	.	C	.	.	.	.	.	.	.	.	.
US03_5_2 (DQ172384.1)	2	*	.	.	.	.	.	.	..	.	.	.	.	.	.	.	.	T	.	.
GB89_2_1 (DQ172365.1)	3	*	.	.	.	.	A	.	..	.	A	.	T	.	.	.	.	.	.	.
GB00_1_1 (DQ172332.1)	4	*	.	A	.	.	A	.	..	.	.	.	.	.	.	.	.	.	.	.
US01_1_2 (DQ172375.1)	5	*	.	.	.	.	A	.	..	.	G	A	.	.	.	.	.	.	.	.
V592 (AY464052.1)	6	.	T	.	.	.	A	.	..	.	.	.	.	.	.	.	T	.	.	.
GB85_1_1 (DQ172359.1)	6	*	T	.	.	.	A	.	..	.	.	.	.	.	.	.	T	.	.	.
09m142 (MN226987)	Not ass	.	.	.	.	.	A	.	..	Start gap	-	-	-	-	-	-	-	-	End gap	.
RacL11 (MF975656.1)	Not ass	A	.	.	.	.	A	.	..	Start gap	-	-	-	-	-	-	-	-	End gap	.
KyA (MF975655.1)	Not ass	A	.	.	.	.	A	.	GC	Start gap	-	-	-	-	-	-	-	-	End gap	.
● 15D59 (MT940243)	Not ass	.	.	.	.	C	A	.	..	Start gap	-	-	-	-	-	-	-	-	End gap	.
● 15Q25-1 (MT940244)	Not ass	.	.	.	.	C	A	.	..	Start gap	-	-	-	-	-	-	-	-	End gap	.
● 16Q4 (MT940245)	Not ass	.	.	.	.	.	A	G	..	Start gap	-	-	-	-	-	-	-	-	End gap	.
● 16Q5 (MT940246)	Not ass	.	.	.	.	.	A	.	..	Start gap	-	-	-	-	-	-	-	-	End gap	.
● 16Q40 (MT940247)	Not ass	.	.	.	.	C	A	.	..	Start gap	-	-	-	-	-	-	-	-	End gap	.
● 18D99 (MT940248)	Not ass	.	.	.	.	C	A	.	..	Start gap	-	-	-	-	-	-	-	-	End gap	.
● 19R166-1 (MT940249)	Not ass	.	.	.	.		A	.	..	Start gap	-	-	-	-	-	-	-	-	End gap	.
● 19R166-6 (MT940250)	Not ass	.	.	.	.		A	.	..	Start gap	-	-	-	-	-	-	-	-	End gap	.
● 19/10/15-2 (MT940251)	Not ass	.	.	.	.		A	.	..	Start gap	-	-	-	-	-	-	-	-	End gap	.
● 19/10/15-4 (MT940252)	Not ass	.	.	.	.		A	.	..	Start gap	-	-	-	-	-	-	-	-	End gap	.
● 19/10/18-2 (MT940253)	Not ass	.	.	.	.		A	.	..	Start gap	-	-	-	-	-	-	-	-	End gap	.
● 19/10/22-1 (MT940254)	Not ass	.	.	.	.		A	.	..	Start gap	-	-	-	-	-	-	-	-	End gap	.

Sequences are aligned with reference to the prototype isolate Ab4 (AY665713.1). Not ass represents the ORF68 not assigned group. Dashes indicate (-) gaps in the alignment, dots (. or ..) indicate the sequence, (●) represents the isolated strain in Korea. Asterisks (*) indicate nucleotides not available in GenBank. Aligned in implemented in BioEdit (v. 7.0.5.3).

**Table 3:** Nucleotide variations alignment of ORF34 of the twelve groups formed from the 12 Korean EHV-1 strains using strain Ab4 as reference.

Accession No.	Group	33	60	62	71	73	81	95	104	110	115	148	149	156	159	197	216	256	257	282	285	303	304	317	380	391	402	405	410	414	422	428	477
Ab4 (AY665713.1)	1	C	T	C	C	G	G	C	T	G	G	A	C	G	A	A	G	G	C	T	A	C	T	C	C	T	C	A	T	T	C	C	G
●16Q4 (MT880905)	1	.	.	.	.	.	.	.	.	.	.	.	.	.	.	.	.	.	.	.	.	.	.	.	.	.	.	.	.	.	.	.	.
●19R166-1 (MT880910)	1	.	.	.	.	.	.	.	.	.	.	.	.	.	.	.	.	.	.	.	.	.	.	.	.	.	.	.	.	.	.	.	.
●19R166-6 (MT880911)	1	.	.	.	.	.	.	.	.	.	.	.	.	.	.	.	.	.	.	.	.	.	.	.	.	.	.	.	.	.	.	.	.
●19/10/15-2 (MT880906)	1	.	.	.	.	.	.	.	.	.	.	.	.	.	.	.	.	.	.	.	.	.	.	.	.	.	.	.	.	.	.	.	.
●19/10/15-4 (MT880907)	1	.	.	.	.	.	.	.	.	.	.	.	.	.	.	.	.	.	.	.	.	.	.	.	.	.	.	.	.	.	.	.	.
●19/10/18-2 (MT880908)	1	.	.	.	.	.	.	.	.	.	.	.	.	.	.	.	.	.	.	.	.	.	.	.	.	.	.	.	.	.	.	.	.
●19/10/22-1 (MT880909)	1	.	.	.	.	.	.	.	.	.	.	.	.	.	.	.	.	.	.	.	.	.	.	.	.	.	.	.	.	.	.	.	.
09m209 (MN226977)	2	.	C	.	.	.	.	.	.	.	.	.	.	.	.	.	.	.	.	.	.	.	.	.	.	.	.	.	.	.	.	.	.
09m34 (MN226975)	2	.	C	.	.	.	.	.	.	.	.	.	.	.	.	.	.	.	.	.	.	.	.	.	.	.	.	.	.	.	.	.	.
17m15 (MN226981)	2	.	C	.	.	.	.	.	.	.	.	.	.	.	.	.	.	.	.	.	.	.	.	.	T	.	.	.	C	.	.	.	.
Buckin.93/2011 (KU206455.1)	3	.	.	T	.	.	.	.	.	.	.	.	.	.	.	.	.	.	.	.	.	.	.	.	.	.	.	.	.	.	.	.	.
NMKT04 (KF644568.1)	3	.	.	T	.	.	.	.	.	.	.	.	.	.	.	.	.	.	.	.	.	.	.	.	.	.	.	.	.	.	.	.	.
89c25 (KF644579.1)	4	.	.	.	.	A	.	.	.	.	.	.	.	.	.	.	.	.	.	.	.	.	.	.	.	.	.	.	.	.	.	.	.
89c105 (KF644577.1)	4	.	.	.	.	A	.	.	.	.	.	.	.	.	.	.	.	.	.	.	.	.	.	.	.	.	.	.	.	.	.	.	.
19m04 (MN226983)	5	.	.	.	.	.	.	.	.	.	.	.	T	.	.	.	.	.	.	.	.	.	.	.	.	.	.	.	.	.	.	.	.
19m05 (MN226984)	5	.	.	.	.	.	.	.	.	.	.	.	T	.	.	.	.	.	.	.	.	.	.	.	.	.	.	.	.	.	.	.	.
KyA (MF975655.1)	6	.	.	.	.	.	.	.	.	.	.	.	.	T	.	.	.	.	.	.	.	.	.	.	.	.	.	.	.	.	.	.	.
NY05 (KF644570.1)	6	.	.	.	.	.	.	.	.	.	.	.	.	T	.	.	.	.	.	.	.	.	.	.	.	.	.	.	.	.	.	.	.
NZA-77 (KT324724.1)	7	.	.	.	.	.	.	.	.	.	.	.	.	T	.	.	.	.	.	.	.	A	.	.	.	.	.	.	.	.	.	.	.
Lincoln.2/2012 (KU206475.1)	7	.	.	.	.	.	.	.	.	.	.	.	.	T	.	.	.	.	.	.	.	A	.	.	.	.	.	.	.	.	.	.	.
●15Q25-1 (MN716797)	7	.	.	.	.	.	.	.	.	.	.	.	.	T	.	.	.	.	.	.	.	A	.	.	.	.	.	.	.	.	.	.	.
●15D59 (MN716796)	7	.	.	.	.	.	.	.	.	.	.	.	.	T	.	.	.	.	.	.	.	A	.	.	.	.	.	.	.	.	.	.	.
●16Q5 (MN716798)	7	.	.	.	.	.	.	.	.	.	.	.	.	T	.	.	.	.	.	.	.	A	.	.	.	.	.	.	.	.	.	.	.
●16Q40 (MN716800)	7	.	.	.	.	.	.	.	.	.	.	.	.	T	.	.	.	.	.	.	.	A	.	.	.	.	.	.	.	.	.	.	.
●18D99 (MN716801)	7	.	.	.	.	.	.	.	.	.	.	.	.	T	.	.	.	.	.	.	.	A	.	.	.	.	.	.	.	.	.	.	.
V592 (AY464052.1)	8	.	.	.	.	.	.	.	.	.	.	.	.	.	.	G	.	.	.	.	.	.	.	.	.	.	.	.	.	.	.	.	.
01c1 (KF644578.1)	9	.	.	.	.	.	.	.	.	.	.	.	.	.	.	.	.	.	T	.	.	.	.	.	.	.	.	.	.	.	.	.	.
90c16 (KF644566.1)	9	.	.	.	.	.	.	.	.	.	.	.	.	.	.	.	.	.	T	.	.	.	.	.	.	.	.	.	.	.	.	.	.
Oxfor.206/2013 (KU206470.1)	10	.	.	.	.	.	.	.	.	.	.	.	.	.	.	.	.	.	.	.	C	.	.	.	.	.	.	G	.	.	.	.	.
T953 (KM593996.1)	10	.	.	.	.	.	.	.	.	.	.	.	.	.	.	.	.	.	.	.	C	.	.	.	.	.	.	G	.	.	.	.	.
717A-82 (KT324733.1)	11	.	.	.	.	.	.	.	.	.	.	.	.	.	.	.	.	.	.	.	.	.	.	.	.	.	.	.	.	C	.	.	.
Jul-45 (KT324725.1)	11	.	.	.	.	.	.	.	.	.	.	.	.	.	.	.	.	.	.	.	.	.	.	.	.	.	.	.	.	C	.	.	.
T-529_10/84 (KF644580.1)	12	T	.	.	A	.	A	.	C	.	C	G	.	.	G	.	A	.	.	C	.	.	.	.	.	.	T	.	.	.	.	.	A
94-137 (KF644575.1)	12	T	.	.	A	.	A	.	C	.	C	G	.	.	G	.	A	.	.	C	.	.	.	.	.	.	T	.	.	.	.	.	A
T-616 (KF644574.1)	12	T	.	.	A	.	A	.	C	.	C	G	.	.	G	.	A	.	.	C	.	.	.	.	.	.	T	.	.	.	.	.	A
5586 (AP012321.1)	Un1	.	.	.	.	.	.	T	.	A	.	.	.	.	.	.	.	.	.	.	.	.	.	.	.	.	.	.	.	.	.	T	.
Cambrid.3/1995 (KU206432.1)	Un2	.	.	.	.	.	.	.	.	.	.	.	.	.	.	.	.	C	.	.	.	.	.	.	.	.	.	.	.	.	.	.	.
00c19 (KF644576.1)	Un3	.	.	.	.	.	.	.	.	.	.	.	.	.	.	.	.	.	.	.	.	.	C	.	.	.	.	.	.	.	.	.	.
Hertf.150/2016 (KY852346.1)	Un4	.	.	.	.	.	.	.	.	.	.	.	.	.	.	.	.	.	.	.	.	.	.	T	.	.	.	.	.	.	.	.	.
RacL11 (MF975656.1)	Un5	.	.	.	.	.	.	.	.	.	.	.	.	.	.	.	.	.	.	.	.	.	.	.	.	C	.	.	.	.	.	.	.
Hampsh.1/2008 (KU206462.1)	Un6	.	.	.	.	.	.	.	.	.	.	.	.	.	.	.	.	.	.	.	.	.	.	.	.	.	.	.	.	.	T	.	.

Dots (.) indicate sequence identity, and (●) represents Korean isolate samples. Un indicates the unassigned group. Aligned in implemented in BioEdit (v. 7.0.5.3).

## Data Availability

Not applicable.

## Supplementary Materials

The following are available online at https://www.mdpi.com/article/10.3390/pathogens10040425/s1, Table S1: GenBank Accession Numbers of selected sequences obtained in this study.
