# Supplementary material for: Evaluation of the Variability of the ORF34, ORF68, and MLST Genes in EHV-1 from South Korea
Source: Pathogens. 2021 Apr 2;10(4):425. doi: 10.3390/pathogens10040425 (PMC8066002; doi:10.3390/pathogens10040425)
Supplement: Supplementary file 1 [file pathogens-10-00425-s001.pdf]

Supplementary Table 1: GenBank Accession Numbers of selected sequences obtained in this study.

Strain	ORF30	ORF33(gB)	ORF34	ORF68
15Q25-1	MT675191	MT559576	MN716797	MT940244
15D59	MT675192	MT559577	MN716796	MT940243
16Q4	MT675193	MT559578	MT880905	MT940245
16Q5	MT675194	MT559579	MN716798	MT940246
16Q40	MT675195	MT559580	MN716800	MT940247
18D99	MT675196	MT559581	MN716801	MT940248
19R166-1	MT675201	MT559582	MT880910	MT940249
19R166-6	MT675202	MT559583	MT880911	MT940250
19/10/15-2	MT675199	MT559584	MT880906	MT940251
19/10/15-4	MT675200	MT559585	MT880907	MT940252
19/10/18-2	MT675197	MT559586	MT880908	MT940253
19/10/22-1	MT675198	MT559587	MT880909	MT940254
